# Oral Fosfomycin Formulation in Bacterial Prostatitis: New Role for an Old Molecule-Brief Literature Review and Clinical Considerations

**DOI:** 10.3390/idr14040067

**Published:** 2022-08-18

**Authors:** Andrea Marino, Stefano Stracquadanio, Carlo Maria Bellanca, Egle Augello, Manuela Ceccarelli, Giuseppina Cantarella, Renato Bernardini, Giuseppe Nunnari, Bruno Cacopardo

**Affiliations:** 1 Department of Biomedical and Biotechnological Science (BIOMETEC), University of Catania, 95123 Catania, Italy; 2 Unit of Infectious Diseases, Department of Clinical and Experimental Medicine, ARNAS Garibaldi Hospital, University of Catania, 95123 Catania, Italy; 3 Department of Biomedical and Biotechnological Science, Section of Pharmacology, University of Catania, 95123 Catania, Italy; 4 Unit of Clinical Toxicology, Policlinico G. Rodolico, School of Medicine, University of Catania, 95123 Catania, Italy; 5 Unit of Infectious Diseases, Department of Clinical and Experimental Medicine, University of Messina, 98122 Messina, Italy

**Keywords:** oral fosfomycin, bacterial prostatitis, urinary tract infections

## Abstract

Bacterial prostatitis infections are described as infections that are difficult-to-treat, due to prostate anatomic characteristics along with clinical difficulty in terms of diagnosis and management. Furthermore, the emergence of multidrug resistant (MDR) bacteria, such as extended-spectrum beta-lactamase (ESBL) producer *Escherichia coli,* also representing the main causative pathogen in prostatitis, poses major problems in terms of antibiotic management and favorable clinical outcome. Oral fosfomycin, an antibiotic commonly used for the treatment of uncomplicated urinary tract infections (UTIs), has been recently evaluated for the treatment of bacterial prostatitis due to its favorable pharmacokinetic profile, its activity against MDR gram-positive and gram-negative bacteria, safety profile, and multiple synergic effect with other antibiotics as well as the low resistance rate. This review addresses fosfomycin pharmacokinetics and pharmacodynamics and discusses the latest clinical evidence on its clinical use to treat acute and chronic bacterial prostatitis in hospitalized patients and in outpatients. As described in several reports, oral fosfomycin may represent a valid therapeutic option to treat susceptible germs commonly causing prostatitis, such as *E. coli* and other *Enterobacterales* as well as *Enterococcus faecium,* even as a first-line regimen in particular clinical settings (patients with previous treatment failure, with allergies or outpatients). Stronger data from further studies, including randomized controlled trials, would be helpful to establish the proper dosage and specific indications.

## 1. Introduction

Male urinary tract infections (UTI) have an overall estimated prevalence between 1.5% and 9%, worldwide [1]. Among them, acute (ABP) and chronic bacterial prostatitis (CBP)—according to the National Institutes of Health (NIH), the classification is also known as category I prostatitis (CIP) and category II prostatitis (CIIP), respectively [2]—are considered cumbersome-to-treat infections owing to limited antibiotic choices and poor drug distribution in prostatic tissue [3]. *Escherichia coli* is considered the main causative agent of both ABP and CBP, although other organisms, including *Enterococcus* spp., *Klebsiella* spp., and *Proteus* spp., are also rising [1,3,4,5,6,7]. Furthermore, the increased prevalence of challenging antibiotic resistant microorganisms, such as extended spectrum beta-lactamases (ESBL) producing *E. coli*, as well as the increasing fluoroquinolone resistance, pose major clinical problems in choosing the appropriate therapy to treat and eradicate UTIs [3,8]. In fact, ESBLs are beta-lactamases able to provide bacterial resistance by hydrolyzing various antibiotics: penicillins, first-, second-, and third generation cephalosporins, as well as aztreonam [9]. For this reason, infections caused by ESBL producer bacteria require the administration of different antibiotic classes, such as fluoroquinolones—that may produce adverse effects due to their known toxicities—or carbapenems, which should be considered last resort drugs that should be spared and can be used only in hospital settings [7] [10]. Alarmingly, the increasing rate of *Enterobacterales* carbapenem resistance (mainly due to carbapenemases’ production), adds additional complications to the treatment of both ABP and CPB [4,8]. An old and well-known ally for the treatment of UTIs sustained by these bacteria is represented by fosfomycin. Since its approval, oral fosfomycin–trometamol formulation has been used to treat uncomplicated cystitis in women provoked by susceptible micro-organisms, due to both fosfomycin’s favorable capacity to gain high bladder concentrations, even after single dose, and to its capacity to not allow bacteria to develop cross-resistance [11,12]. This review briefly reports fosfomycin’s history, pharmacokinetic and pharmacodynamic properties and then the analyses of the data available from scientific literature about fosfomycin–trometamol use in patients with acute and chronic prostatitis. Thanks to its advantageous safety along with its ability to achieve therapeutic concentrations in prostatic secretions, fosfomycin–trometamol results in a valid therapeutic option for the treatment of both ABP and CBP, especially in some settings [3].

## 2. Fosfomycin Pharmacology

Fosfomycin was discovered in 1969 by Hendlin et al. under the name of phosphonomycin. It is a phosphoenolpyruvate analogue isolated in fermentation broths through experimentation with various strains of *Streptomyces* spp., such as, *S. fradiae*, *S. viridochromogenes*, and *S. wedmorensis* [13].

**Figure 1:**
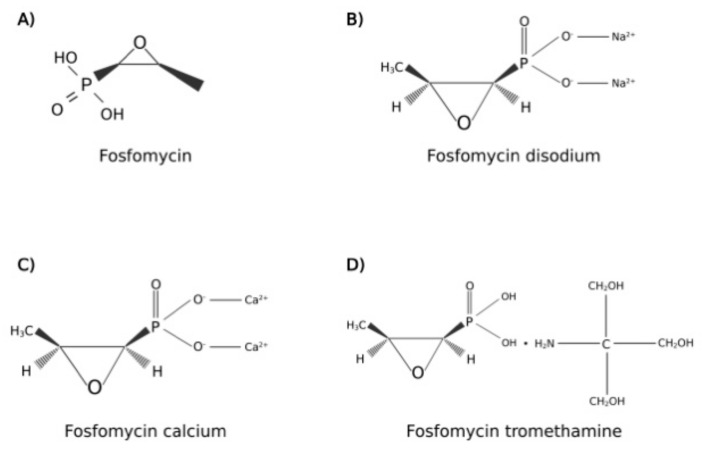
Molecular structures of (**A**) fosfomycin; (**B**) fosfomycin disodium; (**C**) fosfomycin calcium; (**D**) fosfomycin tromethamine. Created with BioRender.com; accessed on 2 August 2022.

The molecular structure of fosfomycin (Figure 1) differs in consideration of drug formulation, indeed, it is available in two oral formulations, fosfomycin calcium and fosfomycin trometamol (also known as fosfomycin tromethamine); this latter is a soluble salt with better bioavailability than fosfomycin calcium. Moreover, an intravenous formulation, fosfomycin disodium, is also commercialized for diverse clinical uses, different from the scope of this review [14].

### 2.1. Mechanism of Action

**Figure 2:**
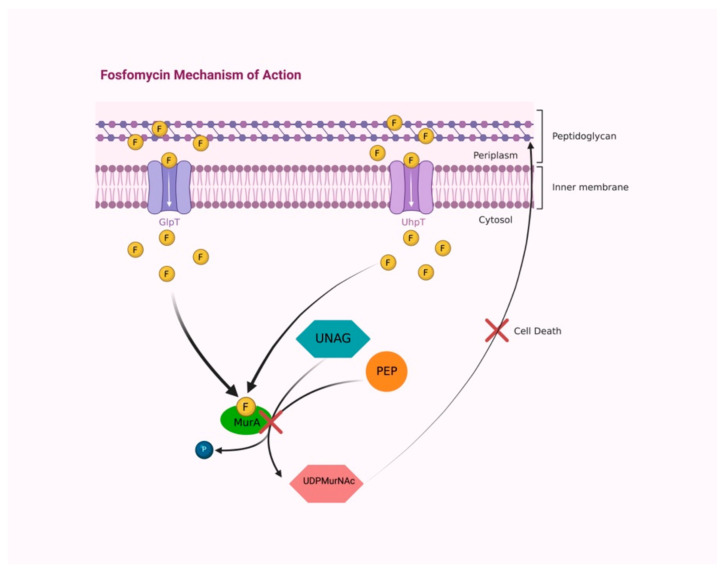
Fosfomycin accesses into the bacterial wall by two transport uptake systems, GlpT and UhpT. In the cytoplasm, fosfomycin covalently binds to the active site of MurA enzyme, preventing the reaction between PEP and UNAG and avoiding UDPMurNAc synthesis, resulting in peptidoglycan building interruption and causing bacterial-cell death. Abbreviations: F, fosfomycin; GlpT, L-alpha- glycerophosphate transport system; UhpT, hexose-6-phosphate transport system; PEP, phosphoenolpyruvate; UNAG, UDP-N-acetylglucosamine; MurA, UDP-N-acetylglucosamine enolpyruvyl transferase; P, phosphate; UDPMurNAc, UDP N-acetylmuramic acid. Created with BioRender.com; accessed on 3 August 2022.

Fosfomycin is a concentration-dependent bactericidal agent with broad bactericidal activity against both gram-positive and gram-negative bacteria, carried out by interfering with the formation of the peptidoglycan precursor UDP N-acetylmuramic acid (UDPMurNAc), the first cytoplasmic step of bacterial cell wall biosynthesis. The enzyme UDP-N-acetylglucosamine enolpyruvyl transferase (MurA) is involved in peptidoglycan biosynthesis by catalyzing the transfer of the enolpyruvyl moiety of phosphoenolpyruvate to the 3′-hydroxyl group of UDP-N-acetylglucosamine (UNAG). Fosfomycin covalently binds the thiol group of a cysteine in the active site of MurA, inactivating it (Figure 2) [15,16].

Two transport uptake systems provide drug access into the bacterial cell, the constitutively functional L-alpha-glycerophosphate transport system (GlpT), induced by glyceraldehyde-3-phosphate, and the hexose-6-phosphate transport systems (UhpT), induced by an extracellular hexose monophosphate inductor, glucose-6-phosphate (G6P) [17]. Its unique mechanism of action-targeting early steps of cell wall synthesis without negatively interfering with other molecules-plays a key role in synergism with other antibiotics, including beta-lactams, aminoglycosides, and fluoroquinolones [14].

### 2.2. Antimicrobial Spectrum

Fosfomycin has broad-spectrum bactericidal activity against staphylococci, enterococci, *Haemophilus* spp., and most enteric gram-negative bacteria. It also has excellent activity against most *E. coli*, including 95.5% of ESBL producing *E. coli* [18]. Conversely, ESBL 025b/B2 *E. coli* strains are resistant to fosfomycin. [18] *Klebsiella* spp. and *Serratia* spp. have higher MICs (from 0.25 to 512 µg/mL for *Klebsiella* spp. with an ECOFF of 128 µg/mL and from 0.5 to 128 µg/mL for *Serratia* spp. with an ECOFF of 32 µg/mL); fosfomycin has activity against only 57.6% of ESBL-producing *Klebsiella* spp [19]. *Pseudomonas aeruginosa* is variably susceptible to fosfomycin, with MICs ranging from 4 to more than 512 µg/mL and with an ECOFF of 256 µg/mL [18]. 

Nearly all isolates of *Acinetobacter baumannii* are resistant to fosfomycin; however, several studies provided evidence of a fosfomycin synergistic effect with amikacin, colistin, and sulbactam to treat *Acinetobacter* infections [20]. Fosfomycin retains excellent in vitro activity against both *E. faecalis* (97.7%) and *E. faecium* (100%) [21]. Moreover, a significant intracellular bactericidal effect was observed for fosfomycin in osteoblast cells infected by *Staphylococcus aureus* [22]. Due to its unique mechanism of action, cross-resistance with other antibiotics is uncommon [23].

### 2.3. Resistance Mechanisms

**Figure 3:**
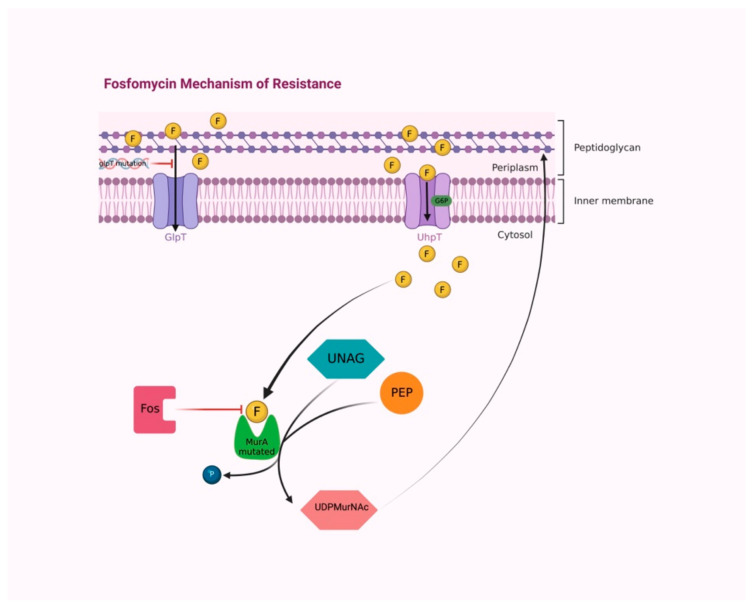
The majority of fosfomycin resistance mechanisms are chromosomally mediated, interfering with the antibiotic transport into bacteria. Mutations of the *glpT* transporter gene cause reduced fosfomycin permeability. The functional G6P-inducible UhpT transport system overrules resistance, maintaining fosfomycin permeability. Cysteine/aspartate substitution in the active site of MurA provokes conformational modification that prevents fosfomycin binding. Fos enzymes inactivate fosfomycin by modifying its molecular structure. Abbreviations: F, fosfomycin; GlpT, L-alpha-glycerophosphate transport system; UhpT, hexose-6-phosphate transport system; G6P, glucose-6-phosphate; PEP, phosphoenolpyruvate; UNAG, UDP-N-acetylglucosamine; MurA, UDP-N-acetylglucosamine enolpyruvyl transferase; P, phosphate; UDPMurNAc, UDP N-acetylmuramic acid; Fos, fos enzymes. Created with BioRender.com; accessed on 4 August 2022.

Mechanisms that confer bacterial resistance to fosfomycin are chromosomally or plasmid-mediated [24]. Most resistance is chromosomally mediated and interferes with the transport of the antibiotic into the bacteria [25]. Concerning the chromosomal one, mutants defective in either the primary entry system via GlpT or in an alternative transport mechanism mediated by UhpT have been demonstrated to reduce fosfomycin intracellular uptake [26]. Nevertheless, the existence of a functional G6P-inducible UhpT transport system often overrules resistance related to mutations in GlpT, resulting in a fosfomycin susceptible phenotype (Figure 3) [27].

Experiments on fosfomycin-resistant strains of *E. coli* allowed for the identification of defects in phosphoenolpyruvate—sugar phosphotransferase system (PTS) and adenylate cyclase activity, resulting in cAMP level reduction and a minor induction of the GlpT transport system [28]. Low levels of cAMP can also occur because of mutations in the *cyaA* gene that codes for adenylate cyclase [29].

Alternatively, fosfomycin may be inactivated by mutations, which allow pyruvyl transferase to distinguish between the substrate phosphoenolpyruvic acid and fosfomycin [30]. Plasmid-mediated *fos*A, *fos*B, *fos*C, *fos*X are metalloenzymes belonging to the glyoxalase superfamily and inactivate fosfomycin by catalyzing its conjugation with glutathione or another nucleophile [31].

Speaking generally, gram-negative (*P. aeruginosa*) bacteria express FosA, a Mn^2+^ and K^+^-dependent glutathione transferase; whilst gram-positive bacteria (*Listeria monocytogenes* and *S. aureus*) produce FosB and FosX, which are a Mg^2+^-dependent L-cysteine thiol transferase and Mn^2+^-dependent fosfomycin-specific epoxide hydrolase, respectively [29,32].

FosC utilizes ATP and exerts its activity by adding to fosfomycin a phosphate group, which also nullifies its antimicrobial properties [33]. These enzymes act by nucleophilic attack on the carbon 1 of fosfomycin, which opens the epoxide ring and inactivates it [34].

Regarding the mechanisms of intrinsic resistance, the lack of fosfomycin effectiveness could result also from the modification of the drug’s target, MurA. Indeed, the substitution of cysteine with aspartate in its active site is established to prevent fosfomycin binding to MurA [35].

### 2.4. Pharmacokinetic Properties

Following oral administration, fosfomycin is rapidly absorbed in the small intestine, where the bioavailability ranges between 34 and 58% for fosfomycin trometamol, whereas it is around 12% for fosfomycin calcium [14]. Apparently, the absorption is not influenced by individual age, although its rate and extent seem to be reduced by food intake (37% fasting versus 30% with food). Additionally, the serum maximum concentration (C_max_) is also higher under fasting conditions, but urinary recovery rates are similar (58% versus 52%) [17].

A pharmacokinetic comparative study, performed in 1988 by Borsa and colleagues in young and elderly adults, demonstrated that fosfomycin trometamol has a higher bioavailability compared to calcium salt. Moreover, at a dose of 2–3 g, the mean peak serum of tromethamine salt was founded to be 2-to-4-fold greater than the one produced by fosfomycin calcium [36].

Those findings probably depend on hydrolyzation and consequently, the inactivation of calcium by gastric acids [37,38,39]. Furthermore, in terms of rate and extent of absorption, a difference between the two oral formulations can be noticed. Two hours after drug administering, the uptake of fosfomycin trometamol was resulted to be six times higher than that of fosfomycin calcium, whereas twelve hours later it was approximately 4 times higher. The binding between antibiotic and plasma proteins is marginal [39].

Following oral administration of fosfomycin trometamol, the mean apparent steady-state volume of distribution (Vss) is 136.1 (±44.1) L. Fosfomycin is not bound to plasma proteins. Oral fosfomycin is distributed to the kidneys, bladder wall, prostate, and seminal vesicles [40].

From 30 to 60% of fosfomycin trometamol is excreted unmodified in the urine vs. 9–18% for the calcium salt. The mean serum elimination half-life (t1/2) of fosfomycin trometamol is estimated at 5.7 h. The area under the concentration time curve (AUC) is 145 to 228 mg h/L [41]. The elimination happens for about 95% through glomerular filtration in the kidneys and no tubular secretion phenomena occur [14]. In addition, oral fosfomycin formulations are subjected to enterohepatic recirculation. Peak urinary concentrations were reported to reach 4000 mg/L and to remain at concentrations >100 mg/L for 48 h [42].

### 2.5. Adverse Drug Reaction

Guidelines about oral fosfomycin administration for uncomplicated UTIs (mostly in women) establish short regimens (single/two 3 g doses), which are unlikely to be responsible for major side effects. Furthermore, oral fosfomycin results to be a well-tolerated drug with only mild gastrointestinal side effects (mainly diarrhea), reported either with a fosfomycin dosage higher than 3 g daily or for prolonged treatment [3]. 

Nonetheless, in clinical trials, the most frequently described adverse events arising in > 1% of the study population are: diarrhea—10.4%, headache—10.3%, vaginitis—7.6%, nausea—5.2%, rhinitis—4.5%, back pain—3.0%, dysmenorrheal—2.6%, pharyngitis— 2.5%, dizziness—2.3%, abdominal pain—2.2%, pain—2.2%, dyspepsia—1.8%, asthenia —1.7%, and rash —1.4%. Other side events developed by patients following fosfomycin administration, with a rate of less than 1% are: abnormal stools, anorexia, constipation, dry mouth, dysuria, ear disorder, fever, flatulence, flu syndrome, hematuria, infection, insomnia, lymphadenopathy, menstrual disorder, migraine, myalgia, nervousness, paresthesia, pruritus, SGPT increased, skin disorder, somnolence, and vomiting [43].

## 3. Rationale for Oral Fosfomycin Administration in Patients with Bacterial Prostatitis

Among antibiotics in clinical use, fosfomycin tromethamine, with a low molecular weight of 138.059 + 121.131 g/mol, is able to reach clinically relevant concentrations in the bladder as well as in the prostatic gland. Its hydrophilicity, together with the negligible protein binding (<5%) and the overall PK/PD profile, allow the antibiotic to reach a bio-availability level of 33–50% with a 2-h concentration of 20–30 mg/L and 2000–2500 mg/L in the serum and in the urine, respectively, after a single oral dose of 3 g [44]. 

Following oral fosfomycin administration in animal models, Fan et al. described the beneficial effects in terms of decreased inflammation, lowering bacterial proliferation and the amelioration of prostatic damage [45]. 

To note, as reported by the European Committee on Antimicrobial Susceptibility Testing (EUCAST), fosfomycin epidemiological cut-offs (ECOFF) for the bacteria most frequently isolated in UTIs range from 4 mg/L for *E. coli* to 8 mg/L for *Proteus mirabilis* and 32 mg/L for *S. aureus* (even lower for MRSA) [46,47], values that are lower than the antibiotic concentration in the urine even after 48 h post antibiotic administration (100–700 mg/L) [44]. Several studies do not endorse fosfomycin administration if its MIC is >4 mg/L, due to the high risk of not achieving efficient intraprostatic concentrations [1].

The reason behind its success in the treatment of UTIs as well as the perioperative prophylaxis of prostate biopsy is due to its unchanged excretion in the urine and almost unchanged renal elimination [44]. On the other hand, this poses a problem in the administration of fosfomycin to patients with compromised renal function.

Although oral fosfomycin administration dates back to the 1970s and some mechanisms of resistances are unknown—as aforementioned—fosfomycin-resistant *E. coli* are still rare, testifying the slow adaptation rate of the bacteria to this molecule, a clear advantage for the physician. Moreover, the mechanism of action of fosfomycin, affecting the early steps involved in bacterial cell wall formation [48], suggests an additive or synergistic action in combination with other antibiotics; in fact, fosfomycin shows important synergistic effects with many other antibiotics, e.g., piperacillin/tazobactam, ceftazidime/avibactam, meropenem, colistin, and daptomycin, as well as linezolid [14,44,49,50,51,52,53,54,55,56,57,58,59].

Some issues can arise in performing fosfomycin susceptibility testing due to the aforementioned fosfomycin mechanism of entry requiring the presence of G6P. Because of that, the gold standard method for *Staphylococci*, *Enterococci*, *Enterobacterales* and *P. aeruginosa* is the agar dilution with the addition of G6P in the medium [60]. This methodology is time and consumables demanding, which is not suitable for every hospital setting. Luckily, more easy and rapid antimicrobial susceptibility testing methods have been produced by different companies and several studies have been performed demonstrating the validity of the disk diffusion and gradient test, as well as automatized methods [61,62,63,64,65,66]. The accuracy of the results depends on the choice of the most appropriate method for the isolate species, and on the strict adherence to the manufacturers’ instructions, as reported by EUCAST [67]. 

Last but not least, when performing the disk diffusion or gradient test, it could be possible to see colonies within the inhibition zone. In accordance with the EUCAST recommendations, these colonies must be ignored. Luckily, to date, there is no correlation between their appearance and the onset of fosfomycin resistance, which remains very low [68,69].

## 4. Oral Fosfomycin in Chronic Bacterial Prostatitis

Bacterial prostatitis infections, both acute and chronic, are challenging to treat infections, due to the poor antibiotic penetration in prostatic tissue. CBP represents a complex setting to deal with, due to the presence of prostatic calcifications acting as a bacterial sanctuary, and the bacterial biofilm formation, which both lead to relapsing infections, persisting symptoms, and treatment failure; because of this, antibiotic treatment for CBP requires longer duration compared to acute forms, resulting in increased resistance due to selective pressure [3].

Bouiller et al. retrospectively described 17 CBP episodes treated with oral fosfomycin, 3 g every 24–48 h, for a mean duration of 5.5 weeks, achieving a microbiological and clinical cure in more than 90% of episodes. Oral fosfomycin was prescribed mostly to treat CBP due to ESBL producing *Enterobacterales* with resistance to fluoroquinolones and cotrimoxazole in patients with underlying urological disorders, which could explain the incidence of recurrences (58%) in that population [1] (Table 1).

Los-Arcos et al. reported 15 complicated cases of CBP, of which 14 were caused by *E. coli* (4 isolates produced ESBL), whilst in one case the identified etiological agent was *K. oxytoca*; 13 patients received 3 g every 72 h of oral fosfomycin and 2 patients received 3 g every 48 h. Overall, treatment duration was 6 weeks, attaining microbiological examination in 8 of 15 cases, whereas a clinical cure was achieved in 7 of 15 cases; 7 cases failed the fosfomycin therapy within a median follow-up period of 29 months; four out of the six patients diagnosed with prostatic calcifications relapsed within six months [70].

Karaiskos et al. described 44 CBP cases treated with oral fosfomycin, 38 of which had a positive culture for gram-negative bacteria (mostly *E. coli*): 10 isolates produced ESBL, 26 cases were MDR, whilst 6 cultures tested positive for *E. faecalis*. All patients received 3 g daily of oral fosfomycin for one week, followed by 3 g every 48 h for 6 weeks and 12 weeks, in 25 patients and in 19 patients, respectively. A microbiological cure was achieved in 86% of the cases at the end of therapy and 77% at 6 months; the clinical cure was 84% at the end of therapy and 80% at 6 months. Fosfomycin failure was observed in 18% of patients, the majority of whom had a MIC > 16 mg/L [71]. 

Almeida et al. described a case of *E. coli* CBP with several relapsing episodes, despite multiple previous antibiotic regimens; the patient was treated with prolonged fosfomycin therapy: 3 g daily for 10 days, then 3 g every 48 h for three months followed by 3 g weekly for 9 months. Due to intraprostatic calcifications, besides fosfomycin therapy, the patient underwent transurethral resection of the prostate (TURP), achieving a clinical cure confirmed after 9 months of post-therapy follow-up [72].

In a case report, Grayson et al., trying to enhance the antibacterial activity towards an ESBL-*E. coli* responsible for a CBP case, which relapsed after carbapenem therapy, administered 3 g twice daily of oral fosfomycin, causing intense diarrhea and leading clinicians to reduce the dosage at 3 g daily. A microbiological and clinical cure was achieved 6 months after the end of therapy [73]. 

Cunha et al. described the case of an ESBL *E. coli* prostatitis in a patient with a penicillin allergy and affected by prostatic hypertrophy; he did not respond to previous therapies with nitrofurantoin, doxycycline, and oral fosfomycin, showing persisting pyuria and bacteriuria as well as persistent positive urine cultures. A prostatic ultrasound revealed intraprostatic calcifications, without abscesses, and a TURP was performed to remove them.

Therefore, a three-week regimen of 3 g daily of oral fosfomycin plus doxycycline 100 mg twice daily was made, fulfilling a sustained microbiological and clinical cure. Of note, the *E. coli* strains isolated resulted in always being susceptible to fosfomycin (MIC < 4 μg/L) [74].

Recently, Denes described a clinical case about a patient, with a medical history of prostatic surgery and urethral stenosis, who was treated with a course of oral fosfomycin (3 g daily for one week followed by 3 g every 48 h for 3 months) to treat a prostatitis due to *E. coli* resistant to fluroquinolones and cotrimoxazole, and achieved a microbiological and clinical cure in the six-month follow-up. Just as in the case of Cunha et al., previous fosfomycin administration did not result in bacteria resistance [75].

Finally, an uncommon case of persistent CBP, in a patient affected by prostatic hypertrophy and ciprofloxacin allergy due to *Raoultella planticola,* was successfully treated by Gian et al. who administer oral fosfomycin, 3 g daily, for three months [76].

The majority of reported CBP patients received oral fosfomycin as an alternative regimen to the standard of care without standardized duration and dosage, achieving microbiological/clinical eradication and a recurrence rate not so different compared with the classic agent (fluoroquinolones). Prostatic calcifications, along with urinary abnormalities, represent the main causes, which led to antibiotic failure and infection recurrence. Oral fosfomycin did not demonstrate a safety concern, except for self-limiting diarrhea, and previous fosfomycin treatments have not been associated with resistance development [1,70,71,72,73,74,75,76].

## 5. Oral Fosfomycin in Acute Bacterial Prostatitis

Due to prostate inflammatory status in ABP, most antibiotics, including fosfomycin, do penetrate the prostatic tissue [79]. Although the pharmacokinetic and pharmacodynamic proprieties of fosfomycin are very favorable and fosfomycin pharmacological proprieties—especially its low protein binding and high lipid solubility [2], which promote its penetration into the lipid-rich prostatic parenchyma—the use of fosfomycin for the treatment of ABP is still neglected. 

Even though oral administration of fosfomycin trometamol does not represent the first choice for the treatment of ABP, it could be useful for outpatients or for those who cannot receive other drugs due to allergies, as well as being an adjuvant of other antibiotics due to its good synergistic effect and the low rate of resistance [12].

Despite the use of oral fosfomycin for the treatment of urinary tract infections and, somewhat, for the management of CBP, which is common, only a few cases reported the efficacy of oral fosfomycin in the treatment of ABP sustained by diverse bacterial species: in two cases the microorganism responsible for the ABP and treated with fosfomycin was an ESBL producing *E. coli* [73,77], whilst *E. faecium* [78] has been reported only once. 

With regards to ESBL *E. coli* cases, of note is the age difference between the two patients, the 30-year-old [77] and 73-year-old [73]. The younger patient was treated with oral fosfomycin for three weeks under a dose regimen of 3 g once daily for the first week, then switched to 3 g once every 48 h for the remaining two weeks [77]. The older patient received 3 g once daily of oral fosfomycin for 16 weeks [73]. In both cases, the regimen was set in response to the development of diarrhea when the clinicians tried to use a higher dose regimen of antibiotic. The pathogen eradication was reached in both the cases, revealing that the efficacy of fosfomycin seems to not be strictly related to the dosage due to its high concentration in the inflamed prostate.

The only ABP caused by a gram-positive bacterium and treated with fosfomycin reported in literature was sustained by an *E. faecium* resistant to ampicillin, chloramphenicol, vancomycin, gentamycin, and ciprofloxacin, but susceptible to nitrofurantoin and quinupristin-dalfopristin. To note, the strain had a high fosfomycin MIC of 64 mg/L, considered as an intermediate susceptibility by the authors, which nowadays represents the cut-off of susceptibility for *E. faecalis* according to the clinical and laboratory standards institute (CLSI) [80]. Due to the 85-year-old patient’s refusal of intra-venous (IV) therapy and due to the scarce penetration of nitrofurantoin into the prostate, he was treated with a trial of prolonged and unconventional dosing of oral fosfomycin equal to 3 g every 3 days for 21 days. The patient’s symptoms resolved after the second dose and the follow-up of two years demonstrated no recurrence of the infection [78].

According to other authors, fosfomycin could be considered as an alternative therapy for quinolone-resistant ABP [81] or in combination with cefoxitin in its IV form for the treatment of ABP caused by fosfomycin susceptible *Enterobacterales*, paying attention to the heart and renal functionality [82].

## 6. Conclusions

Bacterial prostatitis infections have always represented difficult-to-treat infections because of the challenging diagnosis, and it is often laborious due to unclear clinical signs and prostate anatomical characteristics, which make it a cumbersome target to reach for antibiotics and leads to the concept of the infection “sanctuary”. Furthermore, the emergence of MDR microorganisms poses additional therapeutical problems, especially for frail patients, such as the elderly and immunocompromised [83,84,85,86]. 

Several studies demonstrated that oral fosfomycin possesses an interesting pharmacokinetic and pharmacodynamic profile, which allows greater intraprostatic drug concentrations, which are frequently sufficient to achieve bactericidal effects. However, testing methods and MIC evaluation should be always cautiously observed.

The evidence we reported is more representative for CBP than ABP, and mainly regards *E. coli* infections. Fosfomycin has been used in prolonged course regimen, for both ABP and CBP, and its use was often limited to MDR bacteria, previous treatment failure, and intolerance to previous treatments.

Despite the lack of randomized controlled trials and stronger clinical data, which may be a determining factor, fosfomycin could represent a valid therapeutic option to treat prostatitis caused by susceptible germs, perhaps as first-line regimen in particular clinical settings.

## Figures and Tables

**Table 1:** Clinical use of oral fosfomycin in acute and chronic bacterial prostatitis, causative strains, dose regimens, side effects, and outcomes.

Prostatitis Type	Pathogen (n° of Isolates)	Combination Therapy	Fosfomycin Dosage	Adverse Effect	Clinical Cure	Microbiological Cure	Reference
CBP	*E. coli* (12)	3/12	3 g/24–48 h for 5.5 weeks (mean duration)	Diarrhea (4/12)	Yes	8/12	[1]
	*K. pneumoniae* (5)	1/5		-	4/5	3/5	
	*E. coli* (14)	1/14	3 g/48–72 h for 6 weeks	-	7/14	8/14	[70]
	*K. oxytoca*	No			No	No	
	*E. coli* (29)	No	3 g/24 h for the first week, then 3 g/48 h or 3 g/72 h for 6–13 weeks	Diarrhea (4/44)	23/29	23/29	[71]
	*K. oxytoca* (3)				3/3	3/3	
	*K. pneumoniae* (3)				2/3	2/3	
	*P. mirabilis* (2)				2/2	1/2	
	*P. aeruginosa*				No	No	
	*E. faecalis* (6)				5/6	6/6	
	ESBL-*E. coli*	No	3 g/24 h for 9 days, then 3 g/48 h for 3 months and 3 g/weekly for 9 months	Diarrhea during the first week	Yes	Yes	[72]
	ESBL-*E. coli*	No	15 weeks of 3 g once daily; 5 days of 3 g twice daily	Diarrhea with the doubled dose	Yes	Yes	[73]
	ESBL-*E. coli*	Yes	3 g/72 h	-	Yes	Yes	[74]
	*E. coli*	No	3 g/24 h for 1 week, then 3 g/48 h for 3 months	-	Yes	Yes	[75]
	*R. planticola*	No	3 g/48 h for 3 months	-	Yes	Yes	[76]
ABP	ESBL-*E. coli*	No	3 g/24 h for 1 week—3 g/48 h for 2 weeks	Diarrhea during the first week	Yes	Yes	[77]
	ESBL-*E. coli*	Yes	3 g/24 h—3 g/twice daily (5 days) for 16 weeks	Diarrhea with the doubled dose	Yes	Yes	[73]
	*E. faecium*	No	3 g/72 h for 3 weeks	-	Yes	Yes	[78]

## Data Availability

Not applicable.

## Abbreviations

ABP	Acute bacterial prostatitis
ATP	Adenosine triphosphate
AUC	Area under the concentration time curve
CIP	Category I prostatitis
CIIP	Category II prostatitis
CBP	Chronic bacterial prostatitis
CLSI	Clinical and laboratory standards institute
cAMP	Cyclic adenosine monophosphate
ECOFF	Epidemiological cut-off
EUCAST	European Committee on Antimicrobial Susceptibility Testing
ESBL	Extended spectrum beta-lactamases
G6P	Glucose-6-phosphate
UhpT	Hexose-6-phosphate transport systems
IV	Intra-venous
GlpT	L-alpha-glycerophosphate transport system
MRSA	Methicillin resistant *Staphylococcus aureus*
MIC	Minimum inhibitory concentration
MDR	Multidrug resistant
NIH	National Institutes of Health
PD	Pharmacodynamic
PK	Pharmacokinetic
PTS	Sugar phosphotransferase system
TURP	Transurethral resection of the prostate
UDPMurNAc	UDP N-acetylmuramic acid
UNAG	UDP-N-acetylglucosamine
UTI	Urinary tract infections
Vd	Volume of distribution
